# Case of Calculus in the Bladder

**Published:** 1830-10

**Authors:** Baron Heurteloup


### LITHOTRITY.

*Case of Calculus in the Bladder. Operation of Lithotrity successfully performed by* Baron HEURTELOUP. (Communicated by Dr. DOBSON, Surgeon, Greenwich Hospital.)

SAMUEL GUDGE, aged fifty-four, a pensioner of Greenwich Hospital, was received under my care for gonorrhœa, after having been treated in the usual manner for the space of fifteen days. Supposing that his complaint was not simply gonorrhœa, I introduced a sound, and found a large calculus in the bladder, the existence of which the patient would not believe, as he had never suffered the slightest symptom of stone, nor did he feel the least inconvenience from it, in consequence, as we suppose, of its being lodged in a distention of the bladder corresponding to its size, immediately behind the prostate gland: this inference was drawn from its not being felt at all when the man was sounded standing, but, when he was placed in a horizontal position, it fell to the fundus of the bladder, and could be at all times distinctly felt.

The patient being unwilling to submit to the operation of lithotomy, I proposed to him that of lithotrity, with a view of putting that operation to the test. I had previously seen the instruments of Baron HEURTELOUP, and was satisfied in my own mind that they were calculated to perform all that the Baron promised. The man having given his consent, I requested the Baron Heurteloup to sound him, which he did, and also willingly undertook to operate on him gratuitously. Sir Richard Keats, the governor of the hospital, with the laudable view of promoting science and lessening human suffering, kindly sanctioned the operation.

Before the Baron saw the patient, I dilated the lips of the urethra which were contracted at the superior angle, with a bistoury, and ultimately passed a straight bougie (No. 19,) into the bladder.

May 10th. The rectum having been previously emptied by an enema, the Baron proceeded to the operation: having injected the bladder full of warm water, he introduced his instrument with four branches, and soon succeeded in grasping the stone, which he drilled and excavated; the patient suffering no other pain than a desire to void his urine during the operation. After the Baron had withdrawn his instrument, the urine the patient passed was slightly tinged with blood, and much sand or groundstone came away, but no more blood after the first time.

11th. The patient feels well, but complains of some smarting in making water, which still contains sand.

14th. Urine turbid, but contains no sand.

15th. Sounded him: stone feels rough, and seems to be placed with its long axis from side to side. He complains of some pain in the lumbar region. The quantity of detritus collected from the first drilling was half a drachm.

June 3d. The Baron repeated the drilling and perforation with a larger instrument, with only three branches. The process occupied about a quarter of an hour: a considerable quantity of sand came away in the urine, but no blood.

4th. Sounded, but found the stone not broken. The quantity of detritus from the last operation collected, forty-five grains.

10th. The Baron again repeated the operation, but found more difficulty in introducing the instrument, on account of the urethra being rather swollen, as he supposed, from the former operation. He grasped the stone without difficulty; but to perforate it was not so easy, on account of its extreme hardness, which was so great as to resist the virgule, or excavator, and, in taking another presentation of the stone, one of the claws of the instrument got into a former perforation, and it occupied the Baron several minutes before he could get rid of the stone. After this, when the patient emptied his bladder, some coagulum passed, but water injected immediately came away quite clear.

11th. Urine clear, but some soreness and scalding.

12th. Some hard dark pieces of calculus in the urethra, but the stone still continues large. The quantity of detritus collected since the last operation, 122 grains.

25th. Rigors, succeeded by perspiration. I passed a large sound with great ease. He complains of his water scalding him, and it has an offensive smell. The stone feels rougher than at first, and seems to descend more closely to the neck of the bladder. Baron H. came down today, with the intention of operating, but deferred it in consequence of the man's indisposition.

30th. The Baron operated with an instrument which seizes upon the stone with three claws pointed inwards at almost a right angle, and, when thus held, he strikes the stone with a central rod and hammer, by which the claws reduce the size of the stone, exteriorly. The Baron found considerable difficulty in getting rid of the stone from the instrument, in consequence of it, or some portion of it, having got between the central rod and one of the claws. The patient evacuated several pieces of the exterior part of the stone with the water immediately after the instrument was withdrawn.

July 1st. Has passed a large quantity of laminated fragments of the calculus, and complains of the stone feeling very sharp, particularly after making water. Quantity of detritus collected, thirty-four grains.

7th. The Baron came, and drilled the stone in several places; he also used the hammer once. The man complained of more pain than on former occasions. After the instrument was withdrawn, several large and small pieces of detritus came away.

8th. Complains of numbness about the pubis.

11th. Some uneasiness in perineo, and wished the large sound to be introduced, which passed without difficulty.

13th. Mr. Hume, having analyzed the fragments of the calculus, reported it to be lithic acid, and animal matter, probably urea, mixed with it. The quantity of detritus collected, thirty-four grains.

14th. The Baron operated, but did not seem to perforate the stone so much as usual; he hammered the central rod to reduce the stone exteriorly. The process was rendered more tedious and painful than usual, in consequence of some fragments, mixed with fibrine, getting entangled in the claws, and preventing their being perfectly closed when withdrawn.

15th. A large piece of calculus, weighing ten grains, was extracted from the urethra by the forceps; several smaller fragments being discharged.

18th. Passed a large fragment, in which five laminæ were distinctly seen, dark and light coloured alternately.

20th. A small piece of stone was sticking in the urethra, which came away by injecting the bladder full of water by the stomach pump fitted with a catheter. I afterwards introduced a large sound. The quantity of detritus collected, fifty grains.

At two P.M. the Baron operated with a smaller three-branched instrument, which he preferred in consequence of the difficulty he found the last time: he perforated with it in several places. The quantity of detritus collected, thirty-four grains.

29th. The Baron operated on this occasion with the brisecoque, instead of the trois-branches instrument, and I observed that with it he seized the stone more readily, and gave the patient less pain than with the two others, though he did not suffer much from either. The detritus collected was more square, the quantity twenty-one grains.

August 5th. The Baron repeated the use of the brisecoque, with which he twice seized the stone without difficulty, and gave the patient little or no pain. Some detritus came away with the water when the instrument was withdrawn, and the bladder was subsequently washed out with warm water. In the two last operations no blood passed.

10th. No detritus in the urine: a small portion of stone remains in the bladder, which I felt after having injected it full of warm water.

14th. A little piece of calculus still in the bladder, which is too small to communicate the usual feeling to the hand by the sound, though it can be distinctly heard. Some small fragments came away with the urine after sounding, though none had passed for the last five days.

16th. This forenoon, a fragment of the stone having lodged in the urethra, I removed it by the forceps.

At two P.M. the Baron came prepared to use the brisecoque, but, having injected the bladder, could not find the least particle of stone remaining; so there is reason to believe that the fragment which came away this morning was the small piece I heard yesterday.

17th. The urine clear, and the patient quite well. The quantity of detritus collected, forty-nine grains.

The above operations were witnessed, at different times, by upwards of fifty medical gentlemen, all of whom spoke in the highest terms of admiration of the adroitness with which the Baron used his different instruments, and many were greatly surprised at the facility with which he introduced a straight instrument, as large as No. 18, into the bladder. The time that elapsed between the operations might have been much abridged, but I was fearful of exciting inflammation by too early a repetition, and therefore suffered several days to elapse, during which the patient ailed nothing, before I sent for the Baron, who never followed his own inclination as to the time, but always very politely came when requested.

The size of the stone in this man's case was very considerable, which is proved as well by some of the laminated fragments, which are segments of a large oval, as from the quantity of detritus which we have collected, amounting to seven drachms, (this may be seen at the infirmary of Greenwich Hospital,) independently of much which must have escaped at different times whenever he went to stool. Ten days have now elapsed since the last portion of stone was extracted, and the man is in the enjoyment of good health.

As the above case affords a proof of the success of an operation which is nearly new in this country, I consider it may be advantageous to the profession and the community at large to give it publicity.

